# The Secret behind Extreme Hypoxia Tolerance: A “Slow-Growth” Thoracoabdominal Aneurysm

**DOI:** 10.1055/s-0042-1757796

**Published:** 2022-12-20

**Authors:** Carlo Olevano, Giuliano Gagliardi, Mollo Antonio, Santaniello Eugenio, Loris Flora, Emilio Di Lorenzo, Brenno Fiorani

**Affiliations:** 1 Division of Cardiac Surgery, Department of Cardiovascular Surgery, S.G. Moscati Hospital, Avellino, Italy; 2 Division of Cardiac Imaging, Department of Radiology, S.G. Moscati Hospital, Avellino, Italy; 3 Division of Vascular Surgery, Department of Cardiovascular Surgery, S.G. Moscati Hospital, Avellino, Italy; 4 Division of Cardiology, Department of Cardiovascular Surgery, S.G. Moscati Hospital, Avellino, Italy

**Keywords:** thoracoabdominal aneurysm, frozen elephant trunk, tracheobronchomalacia, extreme hypoxemia

## Abstract

A 61-year-old man presented to our institution complaining of back pain. Breathing was comfortable. An arterial blood gas showed extreme hypoxia causing chronic respiratory alkalosis. Further investigations revealed aneurysmal dilatation of the ascending aorta and the Crawford Type II thoracoabdominal aneurysm, with compression of both the left main bronchus and the right pulmonary artery. The patient was managed with a two-stage hybrid surgical approach comprising total arch replacement using the frozen elephant trunk technique followed by endovascular repair.

## Introduction

Extrinsic main airway or pulmonary artery compression due to thoracoabdominal aortic aneurysm (TAAA) mass effect, although documented, is rare. To the best our knowledge, this is the first report of massive aortic aneurysm causing significant extrinsic compression of both the right pulmonary artery and the left main bronchus in a patient presenting with sudden upper back pain yet no respiratory symptoms.

We chose a two-stage hybrid surgical repair approach, consisting of total arch replacement using the frozen elephant trunk (FET) technique, followed by a thoracic endovascular aortic repair (TEVAR) as a less invasive surgical option.

## Case Presentation

A 61-year-old man presented to our emergency department complaining of sudden back pain without respiratory symptoms. On physical examination, he was afebrile, normotensive, and nontachycardic, with oxygen saturation of 42% and a normal respiratory rate of 20 breaths per minute. The arterial blood gas at room air revealed extreme hypoxemia accompanied by chronic respiratory alkalosis (Pa02: 28.9 mm Hg; PaCO _2_ : 28.9 mm Hg; pH: 7.54).

**Fig. 1:**
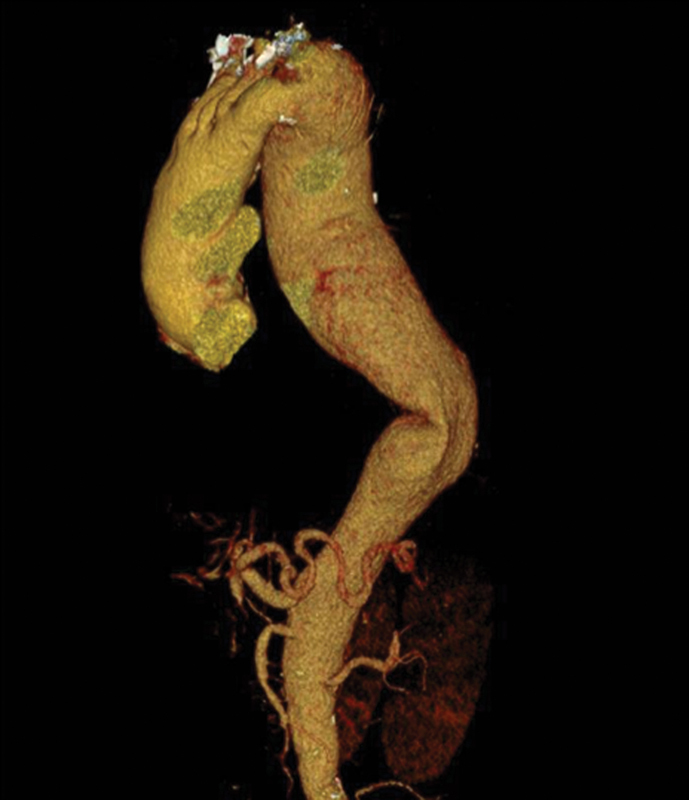
Three-dimensional computer tomography reconstruction of the thoracoabdominal aortic aneurysm.

Computed tomographic scan revealed 5.8-cm aneurysmal dilatation of the ascending aorta and a massive 8.5-cm Crawford's type II thoracoabdominal aneurysm, causing extrinsic compression and near complete occlusion of both the left main bronchus and the right pulmonary artery ( Figs. 1 2 3 ).

**Fig. 2:**
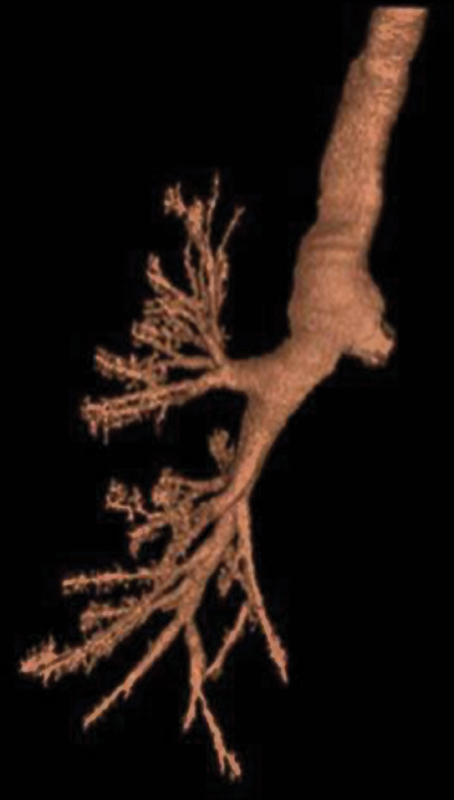
Computed tomography chest airways tree reconstruction showing left-side hypoventilatory phenomenon.

**Fig. 3:**
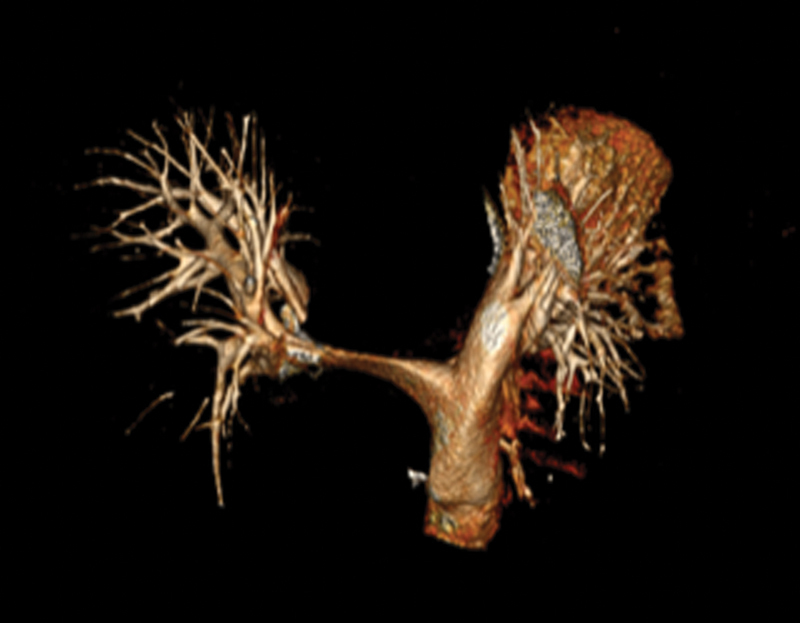
Three-dimensional computed tomography reconstruction of pulmonary artery system showing extreme narrowing of the right pulmonary artery.

**Fig. 4:**
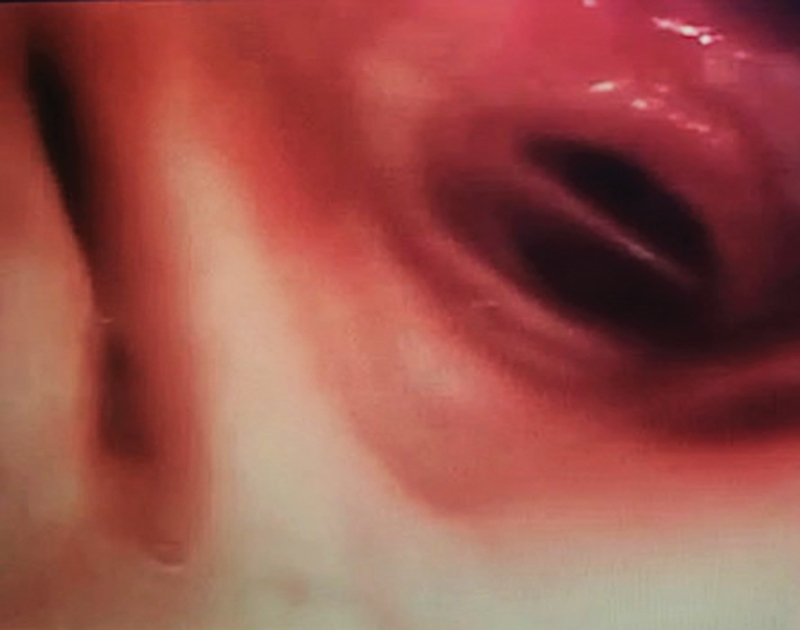
Bronchoscopy image showing expiratory dynamic airways collapse of the left lower lobe bronchus determining partial lung atelectasis.

Further evaluation with flexible fiberoptic bronchoscopy confirmed the anatomical picture ( Fig. 4 ).

The case was discussed within the local heart team, including cardiac surgeons, anesthesiologists, cardiologists, radiologists, and vascular surgeons. A two-stage hybrid surgical approach, comprising total arch replacement using the FET technique and TEVAR, was offered to the patient.

**Fig. 5:**
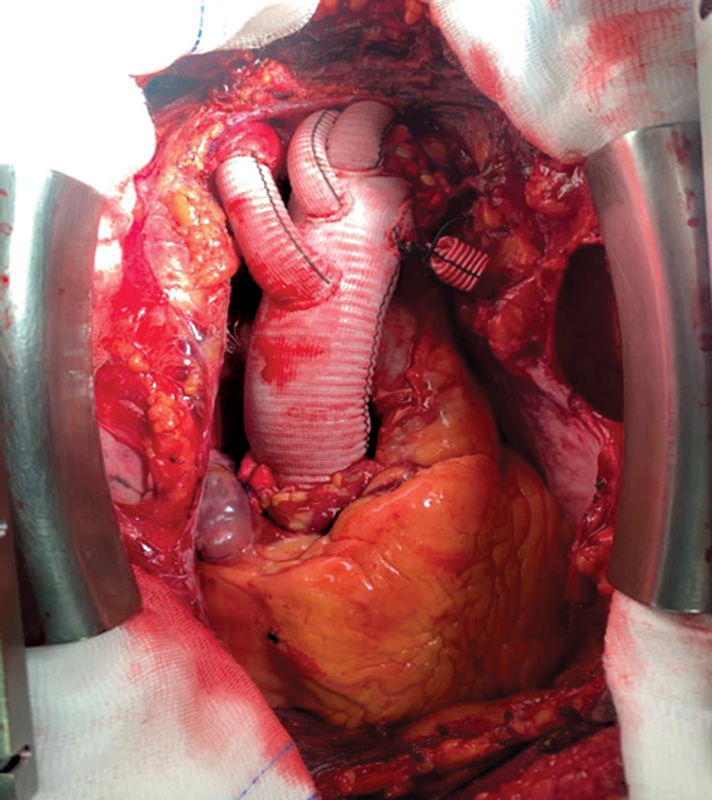
Intraoperative image after Thoraflex (Vascutek Terumo) multibranched hybrid device implantation.

The first stage was performed via median sternotomy. Cardiopulmonary bypass was established via the left axillary artery and the right atrium. Cold Crystalloid cardioplegia (Custodiol, Koehler Chemie, Alsbach-Haenlein, Germany) was used for myocardial protection. Replacement of the aortic arch was performed under moderate hypothermia, circulatory arrest, and selective anterograde cerebral perfusion (SACP). A FET procedure was performed with a 30/32/150-mm Thoraflex (Vascutek Terumo) multibranched hybrid device ( Fig. 5 ).

**Fig. 6:**
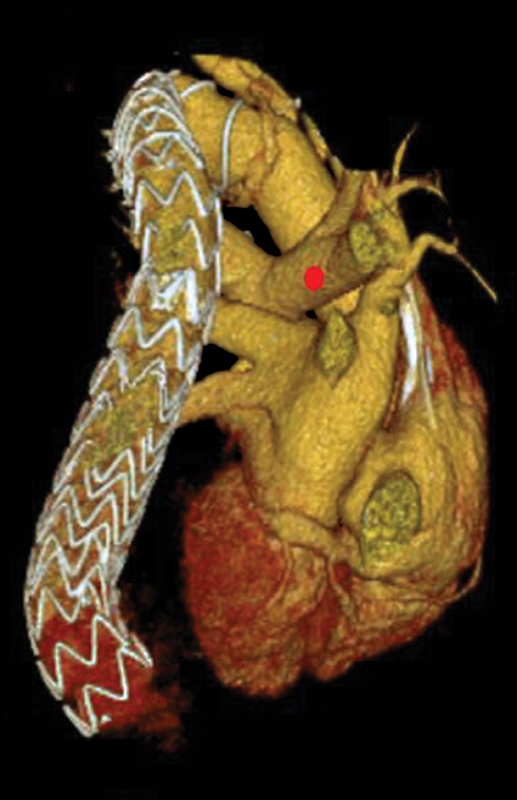
Three-dimensional computed tomography reconstruction showing Thoraflex (Vascutek Terumo) hybrid stented graft and the Valiant thoracic stent in the descending aorta. Postoperative right pulmonary artery relief (red dot).

Two days later, a 40/34/200-mm Valiant thoracic stent graft (with the Captivia delivery system) was deployed through the right femoral artery using the compacted endoprosthesis of the Thoraflex Hybrid graft as a landing zone ( Fig. 6 )

The postoperative course was complicated by surgical tracheostomy due to prolonged intubation and left side bronchomalacia that was successfully treated with positive pressure ventilation and chest physical therapy. Tracheostomy was weaned 42 days after surgery. The patient was ultimately discharged after 8 weeks of hospitalization.

## Discussion

Since the first description of a large TAAA causing airway compression in 1924 1 and pulmonary artery compression four decades later, 2 few cases of thoracic aortic aneurysm causing main airway and/or pulmonary artery extrinsic compression have been documented. To the best of our knowledge, this is the first report of a thoracic aortic aneurysm causing relevant extrinsic compression of both the left main bronchus and the right pulmonary artery. The patient presented with back pain but, surprisingly, normal breathing. The absence of respiratory symptoms in an extremely hypoxic patient is likely due to a TAAA slow growth rate, causing gradual narrowing of the left main bronchus and the right pulmonary artery. Adaptive “body strategies” to tolerate a hypoxic environment include, for example, the ability to alter blood flow distribution, the ability to recruit alternate biochemical pathways, a left-shifted oxyhemoglobin dissociation curve, and a more efficient pulmonary gas exchange. 3

Those “mechanisms” of hypoxia-tolerance in humans have been widely described for professional climbers and for Sherpas, a recently derived Tibetan population resident at high altitude in the Himalayas. 3 4

Tracheobronchomalacia (TBM) is a rare condition characterized by expiratory dynamic airway collapse due to the congenital or acquired weakness of the windpipe. 5 This pathological condition is frequently present in infants and children with congenital heart disease but is uncommon in adults. 6 Treatment of underlying disease is essential to prevent the progression of TBM. In our report, for example, we achieved left main bronchus relief and reversal of left lung atelectasis and vascular compression by aortic aneurism repair surgery and application of postoperative intermittent positive airway pressure and chest physical therapy. However, in cases of severe malacia, more invasive procedures have been described, including stent placement and tracheobronchoplasty. 7 Extrinsic compression of the pulmonary artery by mass effect of thoracic aortic aneurysm is a rare but life-threatening condition, especially when occurring acutely, mimicking pulmonary thromboembolism and causing severe pulmonary hypertension and acute right heart failure. 8 In our report, three-dimensional computer tomography reconstruction showed completely right pulmonary artery relief after surgery ( Fig. 6 , red dot).
